# Does Evidence Permeate All Surgical Areas Equally? Publication Trends in Wound Care Compared to Breast Cancer Care: A Longitudinal Trend Analysis

**DOI:** 10.1007/s00268-012-1599-8

**Published:** 2012-04-19

**Authors:** F. E. Brölmann, M. D. Groenewold, R. Spijker, J. A. van der Hage, D. T. Ubbink, H. Vermeulen

**Affiliations:** 1 Departments of Quality Assurance & Process Innovation, Academic Medical Center, Room A3-503, Meibergdreef 9, P.O Box 22700, 1100 DE Amsterdam, The Netherlands; 2 Department of Surgery, Academic Medical Center, Amsterdam, The Netherlands; 3 Central Medical Library, Academic Medical Center, Amsterdam, The Netherlands; 4 The Netherlands Cancer Institute, Amsterdam, The Netherlands; 5 Amsterdam School of Health Professions, University of Amsterdam, Amsterdam, The Netherlands

## Abstract

**Background:**

Evidence-based decision making has permeated the daily practice of healthcare professionals. However, in wound care this seems more difficult than in other medical areas, such as breast cancer, which has a similar incidence, variety of etiologies, financial burden, and diversity of treatment options. This incongruence could be due to a lack in quantity and quality of available evidence. We therefore compared worldwide publication trends to answer whether research in wound care lags behind that in breast cancer.

**Methods:**

In order to assess the trends in quantity and methodological quality of publications as to wound care and breast cancer treatments, we examined relevant publications over the last five decades. Publications in MEDLINE were classified into seven study design categories: (1) guidelines, (2) systematic reviews (SR), (3) randomized (RCT), and controlled clinical trials (CCT), (4) cohort studies, (5) case-control studies, (6) case series and case reports, and (7) other publications.

**Results:**

We found a 30-fold rise in publications on wound care, versus a 70-fold increase in those on breast cancer. High-quality study designs like SR, RCT, or CCT were less frequent in wound care (difference 1.9, 95 % CI 1.8–2.0 %) as were guidelines; 76 on wound care versus 231 for breast cancer.

**Conclusions:**

Publications on wound care fall behind in quantity and quality as compared to breast cancer. Nevertheless, SR, RCT, and CCT in wound care are becoming more numerous. These high-quality study designs could motivate clinicians to make evidence-based decisions and researchers to perform proper research in wound care.

**Electronic supplementary material:**

The online version of this article (doi:10.1007/s00268-012-1599-8) contains supplementary material, which is available to authorized users.

## Introduction

Every day, surgeons are charged with solving decisional dilemmas while taking care of their patients. Ideally, such choices are based on best available evidence, clinical expertise, and patient preferences. This evidence-based decision making has gradually permeated the daily practice of modern healthcare professionals [1–3] and is endorsed by the U.S. National Institute of Medicine [4]. It is safe to say that nowadays no surgical area is exempt from the obligation to generate and use convincing evidence in the practice high-quality patient care. However, the principle of evidence-based practice has not been implemented equally among all surgical areas [5]. For example, in wound care, evidence-based decision making seems to flourish less than in many other medical areas [6]. The reason for this is unclear, particularly considering the financial impact, prevalence, and effect on quality of life that make wound care a serious health care burden that needs to be relieved by proper evidence 7–11]

A representative illustration of the situation in another surgical area is found in breast cancer. This is a disorder in which huge amounts of money have been invested for research purposes. Although this disorder is obviously different from wounds, it has remarkable similarities in terms of being a surgical disorder characterized by a large diversity of etiologies, treatment options, and outcomes measured [12]. In addition, the lifetime risks of acquiring breast cancer or a (chronic) wound are similar; roughly one of out of every ten subjects [7, 13–18]. Hence, one might think these two disorders deserve equal research efforts and similarly sized bodies of knowledge to enable evidence-based decision making.

Any discrepancy in evidence-based decision making between the areas of breast cancer and wound care could be due to a difference in the amount of convincing evidence available. Such evidence is preferably derived from systematic reviews (SR), randomized (RCT), or controlled clinical trials (CCT) [2]. However, particularly in the realm of wound care, opinion-based articles conclude that the mainstay of evidence seems to consist of noncomparative research designs, which are much more sensitive to bias [6, 12, 19, 20]. This is articulated by frequent appeals in the conclusion of Cochrane systematic reviews: “evidence is weak, so further research is required to validate these findings” [21–25].

We hypothesize that a lack of convincing evidence in wound care forms a barrier for surgeons to practice evidence-based healthcare. Because the quantity and quality of evidence play a crucial role in decision making, it is interesting to know whether and why empirical evidence features more largely in some medical areas than in others. For this reason, we analyzed and compared the worldwide trends as to the quantity and quality of publications regarding wound care and breast cancer, to answer the following question: Is wound care research behind the times in terms of good quality publication output as compared to breast cancer? The answer to our research question could provide surgeons with information about whether high-quality evidence is available for wounds to promote evidence-based practice in wound care to the same degree that applies in breast cancer. This will also help surgeons with clinical and economical decision making to ensure optimum quality of care.

## Methods

We identified all relevant scientific publications over the last 5 decades concerning wound and breast cancer treatments. We did not exclude publication types like letters, editorials, or comments because publication types incorrectly tagged could be missed using search filters [26]. Search strategies were designed in cooperation with a medical information specialist. We searched MEDLINE from 1961 to 2010 by means of two interfaces: OVID for a wide-ranging search of all publication types, followed by PubMed to find particular guidelines. The general search strategies from the Cochrane Wounds Group and the Cochrane Breast Cancer Group were used (see Electronic Supplementary Material). To distinguish the various study designs, these searches were combined with filters available from the BMJ (British Medical Journal) Evidence Centre, the Cochrane Collaboration and Scottish Intercollegiate Guidelines Network (SIGN) (see Electronic Supplementary Material). We did not apply any search limitations such as publication year, type of article, or language.

Subsequently, the selected publications were classified into one of seven study design categories: (1) guidelines, (2) SR, (3) RCT and CCT, (4) cohort studies, (5) case-control studies, (6) case series and case reports, and (7) other publications. Realizing that the available filters for specific study designs are not perfect [26, 27], we validated our search strategy by means of spot-checks of the publications found in both disorders. For this purpose, we randomly chose 100 publications from each study design and in three different 5-year periods to validate the search filter. Titles and abstracts were screened independently by two researchers as to which study design was used and whether this matched the filters used. All search strategies were adapted until the highest number of correct study designs was found with the lowest number of erroneous ones. Adaptations were made by excluding MESH terms like ”*peptic ulcer/” “*colitis, ulcerative/” “*eye infections”, or by adding terms like ”wound$.ti” and “traumatic wound$.ti”.

Finally, PubMed was independently searched by two researchers to find guidelines. These were checked for relevance; i.e., they should address screening, prevention, etiology, pathology, diagnosis, or treatment.

## Data analysis

We calculated how many of the publications found belonged to our predefined publication type categories. The absolute and relative—i.e., in relation to the total in its category—numbers of publications per five years were recorded and plotted as frequency histograms against their publication date. Differences in percentages were calculated including their 95 % confidence intervals (CI).

## Results

### Quantity and quality of publications

Over the last 5 decades we found a total of 145,114 publications on wound care and 217,484 on breast cancer treatment. For wound and breast cancer treatment alike, the majority of publications were classified as “other publications” (65.6 vs. 72.5 %, respectively), as detailed in the paragraph below.

**Fig. 1:**
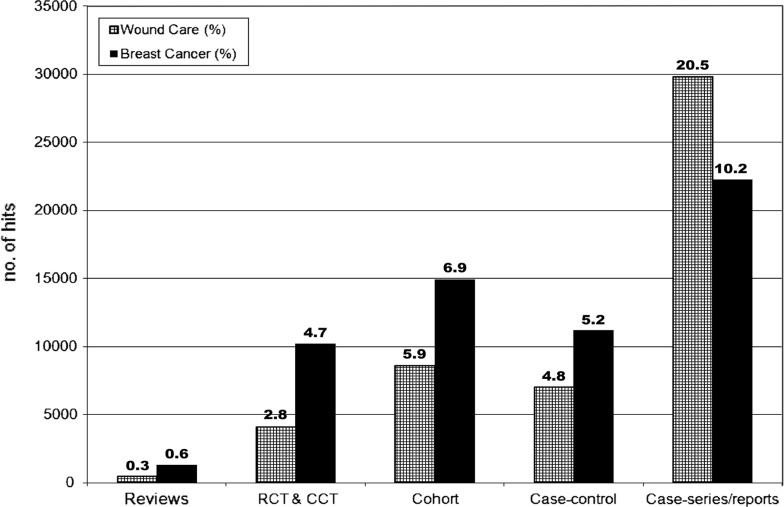
Total numbers of studies found in wound care and breast cancer for each study design (2–6) during the last 5 decades

Differences in quality are illustrated in Fig. 1, which gives an overview of the different study designs (categories 2–6). Studies on wound care were significantly more observational than those on breast cancer (31.2 vs. 22.2 %, respectively; difference 9.0, 95 % CI 8.7–9.3). In addition, the proportion of case series and case reports was significantly higher in wound care (20.5 %) than in breast cancer publications (10.2 %; difference 10.3, 95 % CI 10.0–10.5). Only a very small percentage of the articles (wound care 3.1 %; breast cancer 5.3 %) could be classified as SR, RCT, or CCT, but significantly more on breast cancer (difference 2.16, 95 % CI 2.03–2.29). Thus, over twice as many RCT and CCT were available on breast cancer treatment (10,186) as on wound care (4,061).

### Verification of study categorization: other publications

By means of spot-checks, 100 randomly selected publications in three five-year periods, 1981–1985, 1991–1995, and 2001–2005, were re-categorized by hand, to verify the study type as indicated by the search filter and to check the types of publications grouped in the relatively large category of “other publications.” Over 90 % of the category 2 through 6 study types was found to be correctly classified by the search filters. About three quarters of the spot-check publications were confirmed as “other publications” (Table 1). The remainder, 21.7 % in wounds and 25.7 % in breast cancer, were re-categorized as clinical trial or observational study.

**Table 1:** Result of the spot-checks of category: other publication types

Other publications	Wound care records (%)	Breast cancer records (%)
Publications incorrectly categorized as “other publications”		
Clinical trial	5 (1.6)	3 (1.0)
Observational studies	62 (20.7)	74 (24.7)
Subtotal	65 (21.7)	77 (25.7)
Publications correctly categorized as “other publications		
(Narrative) review	55 (18.3 %)	70 (23.3)
Pilot evaluation		1 (0.3)
Laboratory studies (in vitro)	60 (20.0 %)	46 (15.3)
Animal studies or plant studies	19 (6.3 %)	5 (1.6)
Letter, comment, or editorial	17 (5.7 %)	17 (5.7)
Unknown (e.g., insufficient information available)	80 (26.7 %)	83 (27.7)
Economic evaluation	4 (1.3 %)	
Subtotal	235 (78.3)	223 (74.3)
Total	300 (100)	300 (100)

### Publication trends in time

**Fig. 2:**
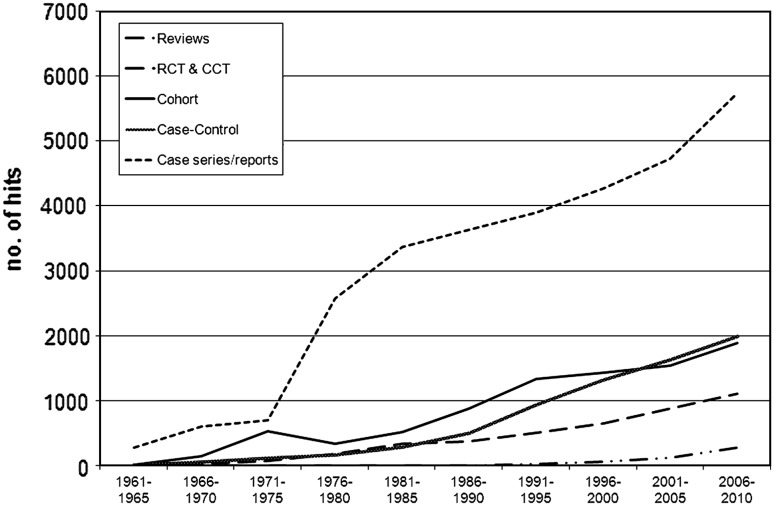
Wound care publication trends by study design

**Fig. 3:**
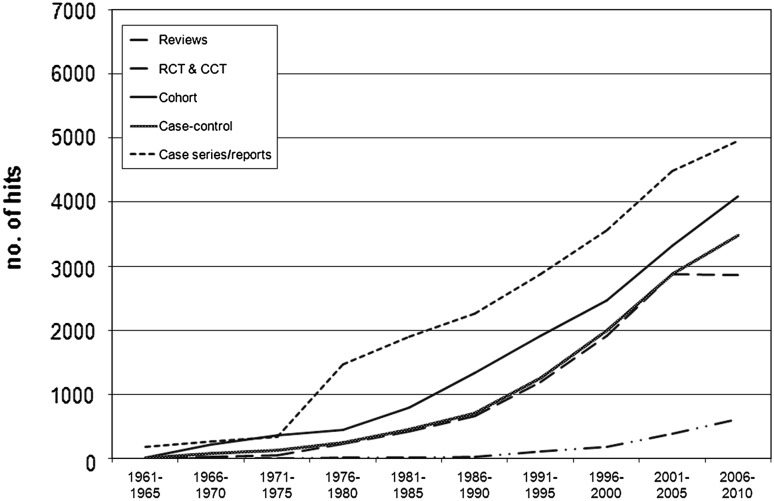
Breast cancer publication trends by study design

Figures 2 and 3 show the number of publications between 1961 and October 2010 in 5-year intervals for wound care and breast cancer. During the past 50 years, breast cancer publications showed a higher number and a quicker growth than wound care publications. In both disorders, the numbers of publications increased substantially. However, for wound care this was an approximately 30-fold increase, whereas for breast cancer it was a 70-fold increase. This trend was more pronounced for the number of trials published, i.e., 800-fold for wound care and 1,700-fold for breast cancer.

### Guidelines

**Fig. 4:**
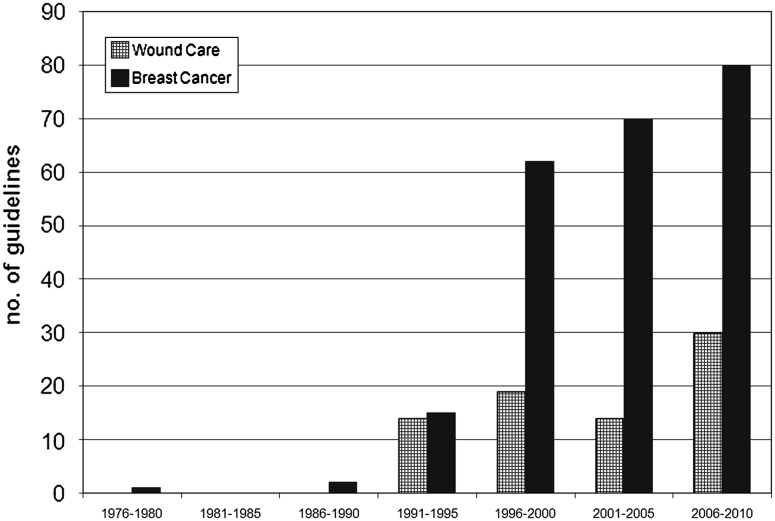
Guideline trends regarding breast cancer and wound care

Of the 211 guidelines found for wound care, only 76 (36 %) guidelines were indeed relevant to wound care. The other guidelines contained a diversity of other medical specialties not related to wounds. In contrast, for breast cancer, 231 (90 %) of the guidelines found were relevant to breast cancer. Figure 4 shows that the number of wound care guidelines increased 5.4 times over the last 5 decades, while breast cancer guidelines showed a 15.4-fold increase over the same period of time. Table 2 shows that guidelines for wound care applied mostly to chronic wounds (68 %), rather than acute wounds (2 %), prevention, diagnosis, or pathology. Table 3 shows a wide variety in terms of screening, diagnostic, and treatment guidelines (73 %), whereas mammography (4 %) and pathology (4 %) guidelines were less published for breast cancer.

**Table 2:** Guideline subjects for wound care

	1961–1965	1966–1970	1971–1975	1976–1980	1981–1985	1986–1990	1991–1995	1996–2000	2001–2005	2006–2010	Total
Acute wounds	0	0	0	0	0	0	0	0	1	1	2 (3 %)
Burns	0	0	0	0	0	0	0	0	2	6	8 (11 %)
SSI	0	0	0	0	0	0	2	3	0	1	6 (8 %)
Chronic wounds	0	0	0	0	0	0	9	14	10	19	52 (68 %)
Other	0	0	0	0	0	0	3	2	1	3	9 (12 %)
Total	0	0	0	0	0	0	14	19	14	30	77 (100 %)

*SSI* surgical site infection

**Table 3:** Guideline subjects for breast cancer

	1961–1965	1966–1970	1971–1975	1976–1980	1981–1985	1986–1990	1991–1995	1996–2000	2001–2005	2006–2010	Total
Screening/diagnostic	0	0	0	1	0	2	7	19	27	22	78 (34 %)
Mammography	0	0	0	0	0	0	2	2	0	5	9 (4 %)
Pathology	0	0	0	0	0	0	2	5	1	1	9 (4 %)
Therapy	0	0	0	0	0	0	1	29	31	30	91 (39 %)
Other	0	0	0	0	0	0	3	7	11	22	44 (19 %)
Total	0	0	0	1	0	2	15	62	70	80	231 (100 %)

## Discussion

The results of our study confirm a rising number of publications for both wound care and breast cancer, which is no different from other areas in medicine. However, the quantity of publications on breast cancer is larger and has a more exponential character in time as compared to wound care. Also, the quality of studies in terms of robust study designs differs in favor of breast cancer. Significantly more clinical trials and fewer case series or case reports have been reported on breast cancer than on wound care.

We are convinced that these findings present a message that is valuable for surgeons. Although there is an inequality in robust knowledge on wound care compared to other areas, sound evidence is available and should be taken into account by surgeons in their decision making. Wound care and wound healing are of great value to all surgical patients, despite the tendency among some surgeons to consider wounds as a mere tailpiece of surgical procedures. This study should be reason to increase awareness among surgeons of available evidence for wounds.

This study is unique in its kind, as it compares trends in quality and quantity of publication output within these two medical areas. Although no classic examples for this kind of bibliometric research are available to mirror our design and outcomes, we assume our results are likely to be valid. This assumption is based on our use of the generally accepted and sensitive search strategies from the Cochrane Wounds Group and the Cochrane Breast Cancer Group, the spot-checks, and the expertise of our medical information specialist. Furthermore, the spot-checks confirmed the reliability of the different filters used to categorize the studies with exception of the remaining group: other study designs.

Some limitations of our analysis need to be mentioned. First, the searches undertaken as part of this study were performed using the MEDLINE database, which is limited to indexed journals. Wound care research is, probably in contrast to breast cancer research, also distributed through non-indexed journals, which could provide an additional number of case series and case report studies that were not captured in this study. Consequently, our search could have underestimated the proportion of case series and case reports, as well as the total number of wound care publications. When comparing the available high-level evidence in terms of systematic reviews, RCT and CCT, such studies are likely to be published on both disorders alike, possibly fostered by positive publication bias. Adding the attractiveness of breast cancer as a research and societal topic, and the proper scientific evaluation that pharmaceutical treatments for breast cancer require before marketing, it is possible that this kind of research receives more funding and attention than does wound care and is therefore easier to publish. This study clearly shows a difference in publication output between the disorders for which funding, publication bias, and demand are all plausible causes of these differences.

Second, we limited our analysis to the last five decades. However, the numbers of publications found before 1960 were negligible and unlikely to influence the results of the observed publication trends. Furthermore, our aim was to study overall publication trends, rather than to give a complete historical overview of publications.

Third, it is important to consider the advantages and limitations of a broad search strategy. Its main advantage is a high sensitivity. As a consequence, however, more hits irrelevant to our medical area appeared in such a search strategy, which may have caused an overestimation of the quantity of publications in both areas. We assumed that the number of irrelevant hits would be equally high in both groups and would therefore not interfere with our conclusions. A further limitation of this search strategy might have been the different search strategies used for each medical area. On the other hand, two researchers (M.G. and F.B.) performed the search independently, and their results were similar.

Fourth, the idea of comparing breast cancer to wound care can be questioned. This comparison might seem far-fetched, as breast cancer is a malignant, potentially life-threatening disease while suffering from a wound is not. However, both are very similar in terms of their widespread occurrence, disease burden, and variation in etiology, treatment options, outcome measures, and patients affected. This should be reason for a similar urgency to generate strong evidence regarding their treatments.

Finally, using the recently developed filters to retrieve guidelines in PubMed, we often found duplicate guidelines regarding the same topic or articles that did not include a guideline at all. Even though the same filter was used, this problem appeared larger in wound care. The wound care guidelines reported in this article could therefore be an underestimation of the problems in wound care research and should be further explored to produce new research questions relevant to patients and clinicians.

Although the field of wound care appears somewhat smaller and publications do fall behind in quantity and quality, our analysis shows that systematic reviews—RCT and CCT—in wound care are being performed and are even on the rise in the last decades. This knowledge helps in building arguments against those who claim it is hard to design, conduct, or apply sound research in wound care [28]. The small number of (evidence-based) guidelines for wound care, especially for acute wounds, revealed a niche that has to be addressed in the near future to help clinicians in evidence-based decision making and to facilitate evidence-based medicine in the wound care area.

## Electronic supplementary material

Below is the link to the electronic supplementary material.

Supplementary material 1 (DOC 51 kb)

Supplementary material 2 (DOC 52 kb)

Supplementary material 3 (DOC 65 kb)
