# Supplementary material for: Does Evidence Permeate All Surgical Areas Equally? Publication Trends in Wound Care Compared to Breast Cancer Care: A Longitudinal Trend Analysis
Source: World J Surg. 2012 Apr 19;36(9):2021–7. doi: 10.1007/s00268-012-1599-8 (PMC3414698; doi:10.1007/s00268-012-1599-8)
Supplement: Supplementary file 1 — Supplementary material 1 (DOC 51 kb) [file 268_2012_1599_MOESM1_ESM.doc]

Wound Care Search strategy in MEDLINE


1.	exp *Peptic Ulcer/ 
2.	exp *Colitis, Ulcerative/ 
3.	exp *Eye Infections/ 
4.	exp *Corneal Ulcer/ 
5.	exp *Dentistry/ 
6.	exp *Tooth Diseases/ 
7.	exp *Oral Ulcer/ 
8.	peptic ulcer$.ti. 
9.	duodenal ulcer$.ti. 
10.	stomach ulcer$.ti. 
11.	corneal ulcer$.ti. 
12.	(ulcer$ adj colit$).ti. 
13.	gastric ulcer$.ti. 
14.	(dental or peridontal).ti. 
15.	aortocaval fistula$.ti. 
16.	arteriovenous fistula$.ti. 
17.	(snake$ or mosquito$).ti. 
18.	fracture$.ti.
19.	mice.ti.
20.	mice.ab.
21.	rats.ti.
22.	rats.ab.
23.	or/1-22
24.	exp Skin Ulcer/ 
25.	exp Leg Ulcer/
	

26.	exp Foot Ulcer/
27.	exp Diabetic Foot/ 
28.	exp Pressure Ulcer/
29.	exp Pilonidal Sinus/
30.	exp Wounds, Penetrating/
31.	exp Lacerations/ or exp Burns/
32.	exp Wound Infection/
33.	exp Bites,human/
34.	exp Wound Healing/
35.	((plantar or diabetic or heel$ or foot or feet or ischaemic or ischemic or venous or varicose or stasis or arterial or decubitus or pressure or skin or leg or mixed or rheumatoid) adj5 (wound$ or ulcer$)).ti.
36.	(bedsore$ or bed sore$).ti.
37.	(pilonidal sinus$ or pilonidal cyst$).ti.
38.	(cavity wound$ or sinus wound$).ti.
39.	((laceration$ or gunshot or stab or stabbing or stabbed or bite$) adj5 wound$).ti. 
40.	(burn or burns or burned or scald$).ti.
41.	(surg$ adj5 wound$).ti.
42.	(surg$ adj5 infection$).ti.
43.	(wound adj5 infection$).ti.
44.	traumatic wound$.ti.
45.	(donor site$ or wound site$).ti.
46.	(skin abscess$ or skin abcess$).ti.
47.	wound$.ti. 
48.	or/24-47
49.	48 not 23
50.	49 not (exp animals/ not (exp animals/ and humans/))
	
