# Supplementary material for: Does Evidence Permeate All Surgical Areas Equally? Publication Trends in Wound Care Compared to Breast Cancer Care: A Longitudinal Trend Analysis
Source: World J Surg. 2012 Apr 19;36(9):2021–7. doi: 10.1007/s00268-012-1599-8 (PMC3414698; doi:10.1007/s00268-012-1599-8)
Supplement: Supplementary file 2 — Supplementary material 2 (DOC 52 kb) [file 268_2012_1599_MOESM2_ESM.doc]

 Breast Cancer Search strategy in MEDLINE


1.	(exp breast neoplasms/ OR exp "neoplasms, ductal, lobular, and medullary"/)
2.	(((exp breast/ OR breast.tw.) NOT ((breast adj milk).ti,ab,sh. OR (breast adj tender$).ti,ab,sh.)) AND (exp neoplasms/ OR exp lymphedema/ OR exp "Analytical, Diagnostic and Therapeutic Techniques and Equipment"/ OR mammograph$.tw.))
3.	(breast adj25 neoplasm$).ti,ab,sh.
4.	(breast adj25 cancer$).ti,ab,sh.
5.	(breast adj25 tumour$).ti,ab,sh.
6.	(breast adj25 tumor$).ti,ab,sh.
7.	(breast adj25 carcinoma$).ti,ab,sh.
8.	(breast adj25 adenocarcinoma$).ti,ab,sh.
9.	(breast adj25 sarcoma$).ti,ab,sh.
10.	(breast adj50 dcis).ti,ab,sh.
11.	(breast adj25 ductal).ti,ab,sh.
12.	(breast adj25 infiltrating).ti,ab,sh.
13.	(breast adj25 intraductal).ti,ab,sh.
14.	(breast adj25 lobular).ti,ab,sh.
15.	(breast adj25 medullary).ti,ab,sh.
16.	exp mastectomy/
17.	exp mammary neoplasms/
18.	(mammary adj25 neoplasm$).ti,ab,sh.
19.	(mammary adj25 cancer$).ti,ab,sh.
20.	(mammary adj25 tumour$).ti,ab,sh.
21.	(mammary adj25 tumor$).ti,ab,sh.
22.	(mammary adj25 carcinoma$).ti,ab,sh.
23.	(mammary adj25 adenocarcinoma$).ti,ab,sh.
24.	(mammary adj25 sarcoma$).ti,ab,sh.
25.	(mammary adj50 dcis).ti,ab,sh.

	

26.	(mammary adj25 ductal).ti,ab,sh.
27.	(mammary adj25 infiltrating).ti,ab,sh.
28.	(mammary adj25 intraductal).ti,ab,sh.
29.	(mammary adj25 lobular).ti,ab,sh.
30.	(mammary adj25 medullary).ti,ab,sh.
31.	exp breast self-examination/ 
32.	(breast adj25 self$).ti,ab,sh. 
33.	(breast adj25 screen$).ti,ab,sh.
34.	exp mammography/
35.	or/1-34
36.	exp silicone/
37.	(cholangocarcinoma* or colonic* or heart or bronchus* or bladder* or airway* or esophageal* or hepatoma* or colon* or pancreas* or renal* or testicular*).ti.
38.	(testis or hepatocellular or lung or pancreatic or pulmonary or colorectal or rectal or thyroid or gastric or orthodontic or ocular or melanoma or gallbladder or oesophagus or liver or laryngeal).ti.
39.	mice.ti.
40.	mice.ab.
41.	rats.ti.
42.	rats.ab.
43.	or/36-42
44.	35 not 43
45.	44 not (exp animals/ not (exp animals/ and humans/))
	
