# Supplementary material for: Does Evidence Permeate All Surgical Areas Equally? Publication Trends in Wound Care Compared to Breast Cancer Care: A Longitudinal Trend Analysis
Source: World J Surg. 2012 Apr 19;36(9):2021–7. doi: 10.1007/s00268-012-1599-8 (PMC3414698; doi:10.1007/s00268-012-1599-8)
Supplement: Supplementary file 3 — Supplementary material 3 (DOC 65 kb) [file 268_2012_1599_MOESM3_ESM.doc]

Medline search strategies for different study designs (created for the OVID online search interface)
(SYSTEMATIC) REVIEW FILTER
1.	("review" or "review academic" or "review tutorial").pt. 
2.	(medline or medlars or embase or pubmed).tw,sh. 
3.	(scisearch or psychinfo or psycinfo).tw,sh. 
4.	(psychlit or psyclit).tw,sh. 
5.	cinahl.tw,sh. 
6.	((hand adj2 search$) or (manual$ adj2 search$)).tw,sh. 
7.	(electronic database$ or bibliographic database$ or computeri?ed database$ or online database$).tw,sh. 
8.	(pooling or pooled or mantel haenszel).tw,sh. 
9.	(retraction of publication or retracted publication).pt. 
10.	(peto or dersimonian or der simonian or fixed effect).tw,sh. 
11.	or/2-10 
12.	1 and 11 
13.	meta-analysis.pt. 
14.	meta-analysis.sh. 
15.	(meta-analys$ or meta analys$ or metaanalys$).tw,sh. 
16.	(systematic$ adj5 review$).tw,sh. 
17.	(systematic$ adj5 overview$).tw,sh. 
18.	(quantitativ$ adj5 review$).tw,sh. 
19.	(quantitativ$ adj5 overview$).tw,sh. 
20.	(quantitativ$ adj5 synthesis$).tw,sh. 
21.	(methodologic$ adj5 review$).tw,sh. 
22.	(methodologic$ adj5 overview$).tw,sh. 
23.	(integrative research review$ or research integration).tw. 
24.	or/13-23 
25.	12 or 24 	RANDOMISED CONTROLLED TRIAL FILTER
1.	exp randomized controlled trials/ 
2.	"randomized controlled trial".pt. 
3.	"controlled clinical trial".pt. 
4.	(random$ or placebo$).ti,ab,sh. 
5.	((singl$ or double$ or triple$ or treble$) and (blind$ or mask$)).tw,sh. 
6.	(retraction of publication or retracted publication).pt. 
7.	or/1-6 
	CASE CONTROL FILTER
1.	exp case-control studies/ 
2.	(case$ and control$).tw. 
3.	1 or 2	
	COHORT STUDY FILTER
1.	exp cohort studies/ 
2.	cohort$.tw. 
3.	controlled clinical trial.pt. 
4.	epidemiologic methods/ 
5.	limit 4 to yr=1960-1988 
6.	or/1-3,5 

	CASE SERIES/REPORT FILTER
1.	(case$ and series).tw. 
2.	case reports.pt. 
3.	(case$ adj2 report$).tw. 
4.	(case$ adj2 stud$).tw. 
5.	or/1-4	
